# Next-Generation Sequencing of Haematococcus lacustris Reveals an Extremely Large 1.35-Megabase Chloroplast Genome

**DOI:** 10.1128/genomeA.00181-18

**Published:** 2018-03-22

**Authors:** Nicholas Bauman, Srividya Akella, Elizabeth Hann, Robert Morey, Ariel S. Schwartz, Rob Brown, Toby H. Richardson

**Affiliations:** a Synthetic Genomics, Inc., La Jolla, California, USA

## Abstract

Haematococcus lacustris is an industrially relevant microalga that is used for the production of the carotenoid astaxanthin. Here, we report the use of PacBio long-read sequencing to assemble the chloroplast genome of H. lacustris strain UTEX:2505. At 1.35 Mb, this is the largest assembled chloroplast of any plant or alga known to date.

## GENOME ANNOUNCEMENT

Haematococcus lacustris (Chlorophyceae) is an algal species of commercial interest due to is ability to accumulate high levels of the red carotenoid astaxanthin, which is used in fish feed, cosmetics, and nutraceuticals (1–3). Efforts to increase Haematococcus productivity by targeting transformation to both nuclear and chloroplast genomes (4, 5) have been limited by the lack of high-quality genome assemblies. The use of PacBio single-molecule real-time (SMRT) sequencing has gained traction recently for improving genome assemblies, including for algal chloroplasts, due to the long reads that are generated (6, 7). Floydiella and Volvox species have some of the largest chloroplasts known among members of the Viridiplantae at ∼500 kb (8, 9); previously, the largest known chloroplast (1.13 Mb) of any lineage belonged to the red alga Corynoplastis japonica (10).

H. lacustris UTEX:2505 was obtained from the Culture Collection of Algae at the University of Texas and grown in optimal Haematococcus medium (11). Total DNA was extracted using a modified cetyltrimethylammonium bromide method (12). Purified DNA was converted to a SMRTbell library according to the manufacturer’s instructions (PacBio, Menlo Park, CA, USA) and size selected with a 10-kb cutoff using Blue Pippin (Sage Science, Beverly MA, USA). Twenty-four SMRT cells were sequenced using the Pacific Biosciences RS II platform, providing 13.6 Gb of mapped reads. The chloroplast genome was assembled *de novo* using the Hierarchical Genome Assembly Process (HGAP) version 2 algorithm (13), yielding a single contig. Over 91,000 subreads, with a mean subread length of 8,900 bp, went into the assembly of the chloroplast genome. GeneMarkS version 4.17 (14) was used to generate gene predictions *ab initio*. Gene annotations were generated through the Synthetic Genomics, Inc. (La Jolla, CA, USA) proprietary Archetype annotation pipeline, as previously described (15).

The circular closed chloroplast genome was assembled into 1.352 Mb at >500× coverage, containing the small single-copy (SSC) and long single-copy (LSC) regions, as well as two inverted repeat (IR) regions, and having an overall G+C content of 50%. While the total size of the chloroplast genome is very large, the combined size of its coding regions is ∼110 kb, including 125 protein-coding genes and 12 tRNAs. Evidence for both *cis-* and *trans*-splicing events, features seen in other algae (16), is found throughout the chloroplast genome. A total of 139,006 repeats were found and classified into 34,710 families within the chloroplast. The repeats ranged from 42 to 104 bp, with the two most prominent repeats having a length of 43 bp. The copy numbers of each repeat ranged from 5 to 270 and were highly skewed toward intergenic regions throughout the chloroplast. Another highly complex and large (>525 kb) chloroplast genome belonging to Volvox caterii (9) contains large numbers of short repetitive DNA sequences, an issue that has hindered the completion of a full chloroplast genome assembly of that species to date. We believe that this work shows that long-read sequencing with PacBio SMRT technology (or another such platform) can be beneficial to assembling complex and repetitive organellar genomes in addition to complex nuclear genomes. Additionally, the size and abundance (∼50% of total DNA) of this plastid genome mean that more coverage is required to obtain a good nuclear genome for the genus Haematococcus.

### Accession number(s).

The assembled H. lacustris UTEX:2505 chloroplast genome has been deposited at GenBank under the accession number MG677935.
